# Supplementary material for: Antimicrobial Resistance and Molecular Characterization of Methicillin-Resistant Staphylococcus aureus Isolated from Slaughtered Pigs and Pork in the Central Region of Thailand
Source: Antibiotics (Basel). 2021 Feb 19;10(2):206. doi: 10.3390/antibiotics10020206 (PMC7922250; doi:10.3390/antibiotics10020206)
Supplement: Supplementary file 1 [file antibiotics-10-00206-s001.pdf]

antibiotics

MDPI

Suppementary Materials

Table S1. Prevalence of MRSA among different sources.

head	No. of samples		*p*-value
	MRSA Positive	MRSA Negative	
Slaughterhouse in 2017	4	98	0.352
Slaughterhouse in 2018	7	95	
Market in 2017	28	29	0.361
Market in 2018	24	35	
Total 2017	32	127	0.845
Total 2018	31	130	
Slaughterhouse	11	193	<0.001*
Market	52	64	
Market D	1	12	0.035*
Market E	36	39	
Market F	7	5	
Market G	8	8	
Slaughterhouse A	8	60	0.008*
Slaughterhouse B	3	65	
Slaughterhouse C	0	68	

* The *p*-value less than 0.05 was considered statistically significant.

Table S2. Antimicrobial resistance patterns of MRSA isolates from pig and pork.

Antimicrobial resistance pattern.

AMP OXA FOX CHL CLI ERY CIP ENR GEN TET SXT VAN

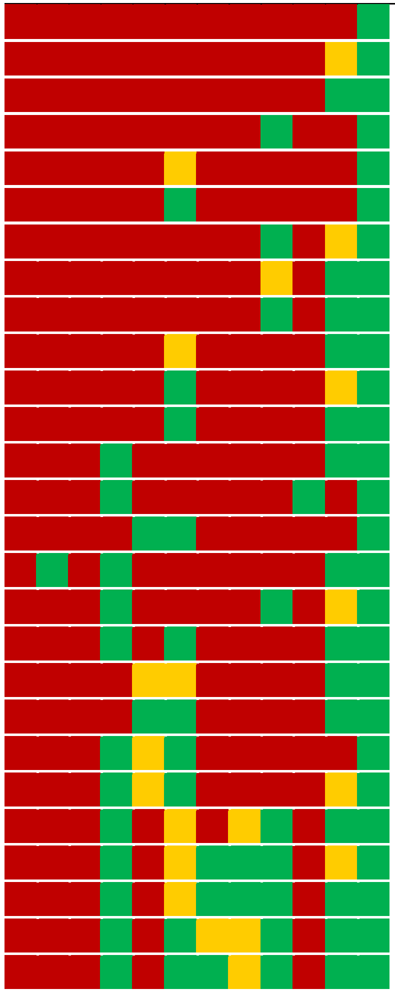

Total of isolates	Sources (No. of isolates): Slaughterhouse	Sources (No. of isolates): Market	Genotype (No. of isolates): ST9 -SCC*mec* IX	ST398 -SCC*mec* V	ST9 -SCC*mec* NT	ST779 -SCC*mec* IV	ST5639 -SCC*mec* IX
5	B (2)	E (3)	5	-	-	-	-
3	A (1)	E (2)	3	-	-	-	-
8	A (3)	E (5)	8	-	-	-	-
2	0	E (2)	2	-	-	-	-
2	0	E (2)	2	-	-	-	-
3	0	E (3)	3	-	-	-	-
1	0	E (1)	1	-	-	-	-
1	0	E (1)	-	-	1	-	-
1	0	E (1)	-	-	1	-	-
3	0	E (1), G (2)	3	-	-	-	-
1	A (1)	0	1	-	-	-	-
7	B (1)	E (3), G (3)	6	-	-	-	1
1	0	F (1)	1	-	-	-	-
1	0	D (1)	1	-	-	-	-
2	0	G (1), E (1)	2	-	-	-	-
1	0	F (1)	1	-	-	-	-
1	0	E (1)	-	1	-	-	-
2	A (1)	F (1)	2	-	-	-	-
1	0	G (1)	1	-	-	-	-
1	0	G (1)	1	-	-	-	-
1	0	F (1)	1	-	-	-	-
1	A (1)	0	1	-	-	-	-
1	A (1)	0	-	1	-	-	-
3	0	E (2), F (1)	-	3	-	-	-
1	0	E (1)	-	1	-	-	-
1	0	F (1)	-	1	-	-	-
1	0	F (1)	-	1	-	-	-

	3	0	E (2), F (1)	-	3	-	-	-
	7	0	E (6), F (1)	-	7	-	-	-
	1	0	E (1)	-	-	-	1	-
**Total**	**67**	**11**	**56**	**45**	**18**	**2**	**1**	**1**

Abbreviation: AMP, ampicillin; OXA, oxacillin; FOX, cefoxitin; CHL, chloramphenicol; CLI, clindamycin; ERY, erythromycin; CIP, ciprofloxacin; ENR, enrofloxacin; GEN, gentamicin; TE, tetracycline; SXY, sulfamethoxazole/trimethoprim; VAN, vancomycin

Red, resistant; Yellow, intermediate; Green, susceptible; NT, nontypeable; ST5639; novel ST found from Market E in 2018

Table S3. Prevalence of antimicrobial resistance of MRSA isolated in slaughterhouse and market located in Pathumthani province, Thailand in 2017 and 2018.

Class/ Antimicrobial agents	No. (%) of antimicrobial resistance of MRSA isolates						
	Slaughterhouse (*n* = 30)			Market (*n* = 102)			Total (*n* = 67)
	2017 (*n* = 4)	2018 (*n* = 7)	Total (*n* = 11)	2017 (*n* = 30)	2018 (*n* = 26)	Total (*n* = 56)	
**Penicillin**							
**AMP**	4 (100)	7 (100)	11 (100)	30 (100)	26 (100)	56 (100)	67 (100)
**OXA**	4 (100)	7 (100)	11 (100)	30 (100)	25 (96.2)	55 (98.2)	66 (98.5)
**Cephem**							
**FOX**	4 (100)	7 (100)	11 (100)	30 (100)	26 (100)	56 (100)	67 (100)
**Phenicol**							
**CHL**	3 (75.0)	5 (71.4)	8 (72.7)	15 (50.0)	19 (73.1)	34 (60.7)	42 (62.7)
**Lincosamide**							
**CLI**	3 (75.0)	7 (100)	10 (90.9)	27 (90.0)	23 (88.5)	50 (89.3)	60 (89.6)
**Macrolide**							
**ERY**	3 (75.0)	3 (42.9)	6 (54.5)	10 (33.3)	9 (34.6)	19 (33.9)	25 (37.3)
**Fluoroquionolone**							
**CIP**	4 (100)	7 (100)	11 (100)	16 (53.3)	23 (88.5)	39 (69.6)	50 (74.6)
**ENR**	4 (100)	6 (85.7)	10 (90.0)	16 (53.3)	23 (88.5)	39 (69.6)	49 (73.1)
**Aminoglycoside**							
**GEN**	4 (100)	6 (85.7)	10 (90.9)	10 (33.3)	23 (88.5)	33 (58.9)	43 (64.2)
**Tetracycline**							
**TE**	4 (100)	7 (100)	11 (100)	30 (100)	25 (96.2)	55 (98.2)	66 (98.5)
**Folate partway -intibitor**							
**SXT**	2 (50.0)	0	2 (18.2)	6 (20.0)	8 (30.8)	14 (25.0)	16 (23.9)
**Glycopeptide**							
**VAN**	0	0	0	0	0	0	0

Abbreviation: AMP, ampicillin; OXA, oxacillin; FOX, cefoxitin; CHL, chloramphenicol; CLI, clindamycin; ERY, erythromycin; CIP, ciprofloxacin; ENR, enrofloxacin; GEN, gentamicin; TE, tetracycline; SXY, sulfamethoxazole/trimethoprim; VAN, vancomycin.

Swab inner wall of pig nose using Culture swab™/ 10 g of pork

↓ Kept in ice box and transport to laboratory within 24 h

Inoculate a swab and pork into 3 ml and 90 ml, respectively, of Trypticase soy broth (contain ceftizoxime, aztreonam, 6.5% NaCl)

↓ Incubate at 37°C, 24 h

Streak the enrich culture on ORSAB agar (oxacillin resistance screening agar base)

↓ Incubate at 37°C, 24 - 48 h

Subculture 3 of representative single colony colored blue on Trypticase soy agar

↓ Incubate at 37°C, 24 h

Confirmed identify on mannitol salt egg-yolk agar, DNAase agar, Coagulase test, antimicrobial susceptibility test

↓

Molecular confirmation tests: 16s rRNA Gene, *mec*A gene

↓

Streak positive MRSA isolates (pure culture) onto a small section of TSA agar plate

↓ Incubate at 37°C, 24 h

Suspend all bacterial grown on TSA into 1ml of 10% skim milk

↓

Store at -80°C

Figure S1. Isolation and Identification for methicillin-resistance *Staphylococcus aureus* (MRSA).
